# An integrated cytomorphology and sequencing analysis for hepatosplenic alpha/beta T‐cell lymphoma

**DOI:** 10.1002/jha2.667

**Published:** 2023-03-06

**Authors:** Hsin‐Yu Lu, Chien‐Chin Lin, Tai‐Chung Huang

**Affiliations:** ^1^ Department of Internal Medicine National Taiwan University Hospital and National Taiwan University College of Medicine Taipei Taiwan; ^2^ Department of Laboratory Medicine National Taiwan University Hospital Taipei Taiwan

**Keywords:** clonal analysis, flow cytometry, morphology, T‐cell lymphoma

1

A 47‐year‐old male with a past medical history of diabetes mellitus under the control of oral hypoglycemic medications developed nausea and intermittent fever for weeks. Physical examination showed splenomegaly (4 cm below the costal margin) without significant lymphadenopathy. Laboratory data showed pancytopenia (Hemoglobin: 11 g/dl, White blood cells: 1.53 k/μl, Platelet: 47 k/μl), elevations of liver function tests (totalbilirubin: 1.38 mg/dl, direct bilirubin: 0.65 mg/dl, alanine transaminase (ALT): 56 U/L, aspartate transaminase (AST): 103 U/L, gamma‐glutamyl transferase (GGT): 515 U/L, alkaline phosphatase (ALP): 260 U/L) and serum lactate dehydrogense (LDH) level (311 U/L, 1.1X). Flow cytometry of peripheral blood revealed that the abnormal lymphoid cells accounted for 4.3% of total nucleated cells, larger‐than‐normal lymphocytes, with positive expression of CD2, CD3, CD7, CD8, CD56 and negative for CD5 and CD45. Bone marrow (BM) aspirate revealed abundant lymphoma cells, med‐ to large‐sized, with pleomorphic nuclei, prominent nucleoli, and coarse cytoplasmic granules (Figure 1). BM biopsy showed hypercellularity and the CD3+ lymphoma cells were negative for CD45, CD34, CD117, TdT, CD79a, and Epstein‐Barr virus‐encoded RNA (EBER) staining, with the pattern of sinusoidal infiltration (Figure S1). BM flow cytometry gating on the lymphoma cells confirmed the positive expression of CD2, CD3, CD7, CD8, CD56, T‐cell receptor (TCR) alpha‐beta chains, perforin, granzyme B, and negative for CD34, CD4, CD5, and TCR gamma‐delta chains (Figure 2), consistent with natural killer (NK)/T lymphoma involvement. Cytogenetic analysis of the bone marrow was normo‐karyotypic. Fluorodeoxyglucose‐positron emission tomography (FDG‐PET) scans revealed hypermetabolism in hepatosplenomegaly (maximum standardized uptake value (SUVmax) = 7.72 in spleen), bilateral cervical nodes, right supraclavicular nodes, right paratracheal nodes (SUVmax = 4.64), L4 (SUVmax = 5.03) and T9 vertebral bodies (SUVmax = 6.3) (Figure 3). Next‐generation sequencing of BM TCR gamma gene VJ‐recombination was indicative of monoclonality of T cells (59.52% of total reads) (Figure 4). This hepatosplenic T‐cell lymphoma, alpha‐beta type, Ann Arbor stage IV was thus diagnosed. He received three cycles of chemotherapy L‐CHOEP (L‐asparaginase, cyclophosphamide, doxorubicin, vincristine, etoposide, prednisolone) followed by a myeloablative matched sibling peripheral stem cell transplant. He remained free from relapse 14 months post‐transplant with a normal hemogram and normal‐metabolic FDG‐PET scans.

**FIGURE 1:**
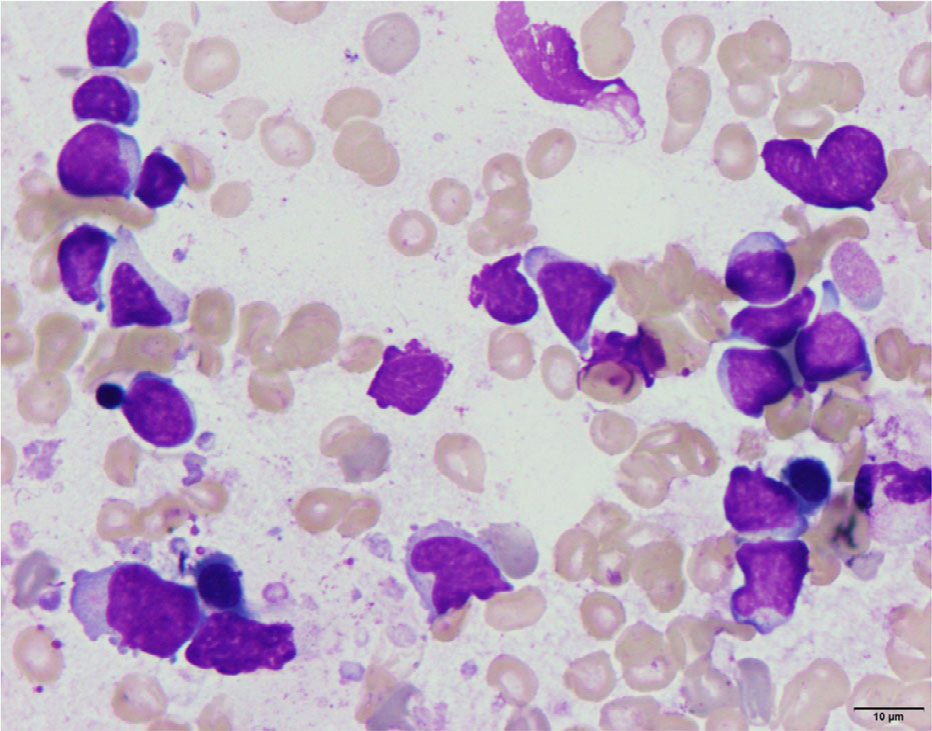
Morphology of the lymphoma cells in the bone marrow (BM) smear, Liu stain, 1000x.

**FIGURE 2:**
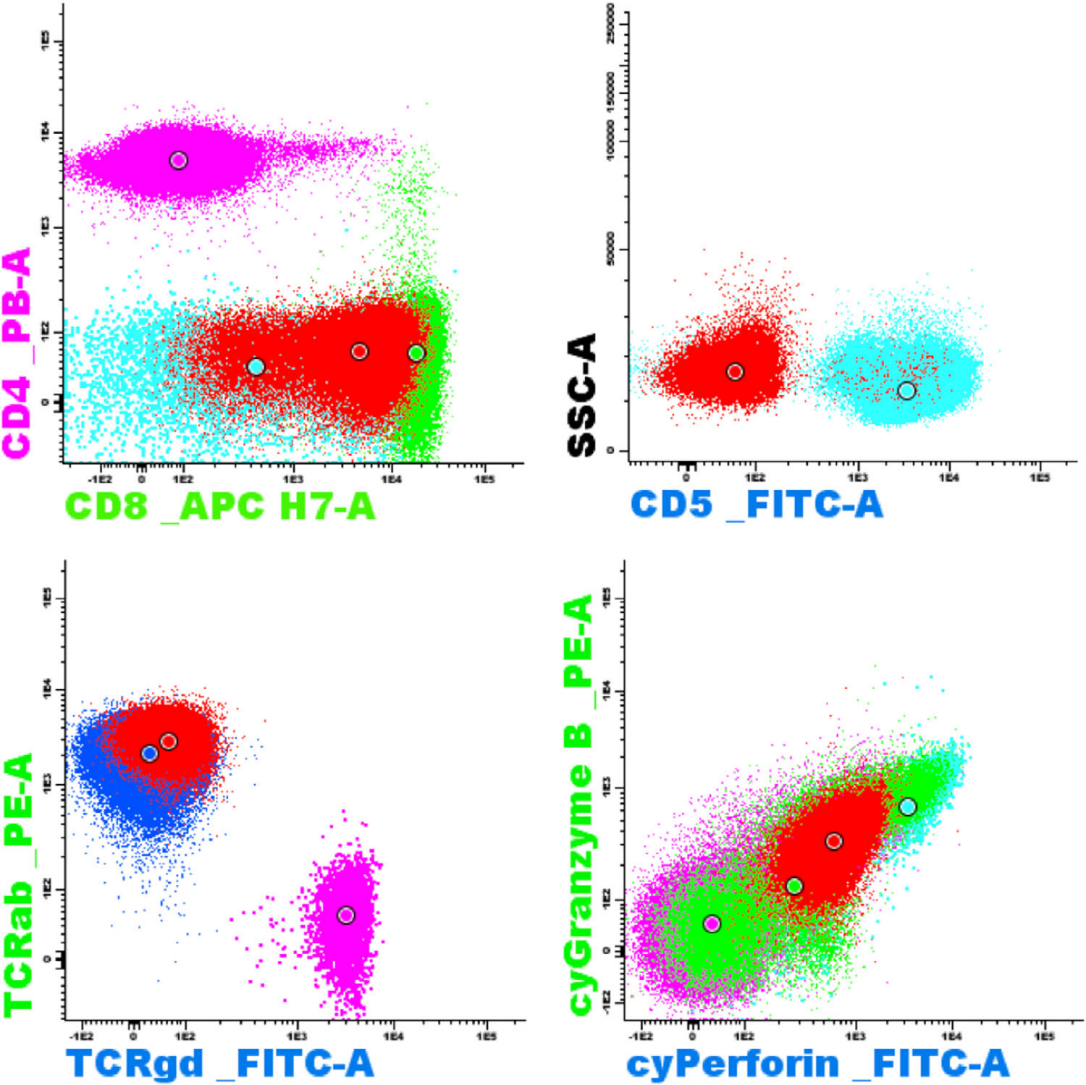
Selected flow cytometry marker expressions of the lymphoma cells (red color).

**FIGURE 3:**
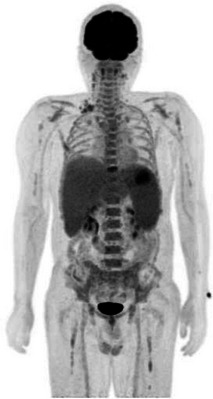
The diagnostic FDG‐PET scans of the patient.

**FIGURE 4:**
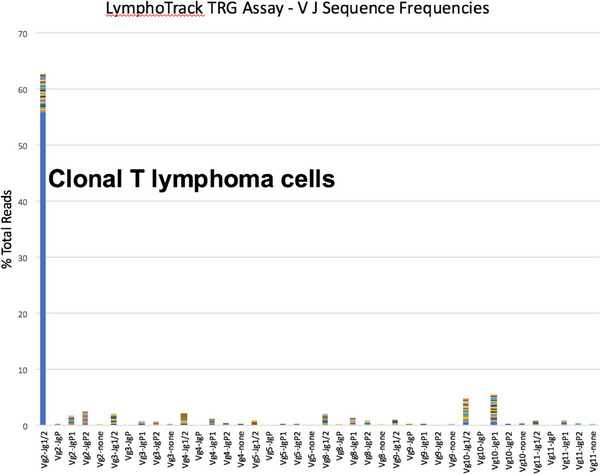
T‐cell receptor (TCR) gamma gene VJ recombination sequence analyses.

## CONFLICT OF INTEREST STATEMENT

The authors declare no conflict of interest.

## PATIENT CONSENT STATEMENT

We obtained informed consent from the patient.

## ETHICS STATEMENT

We have IRB approval from Research Ethics Committee A National Taiwan University Hospital with IRB number: 201910033RSB (Case number: 01–0019).

## Supporting information

Supporting Information

## Data Availability

Data are available in the article's Supporting Information.

