# Supplementary figures and images for: An integrated cytomorphology and sequencing analysis for hepatosplenic alpha/beta T‐cell lymphoma
Source: EJHaem. 2023 Mar 6;4(2):505–7. doi: 10.1002/jha2.667 (PMC10188490; doi:10.1002/jha2.667)

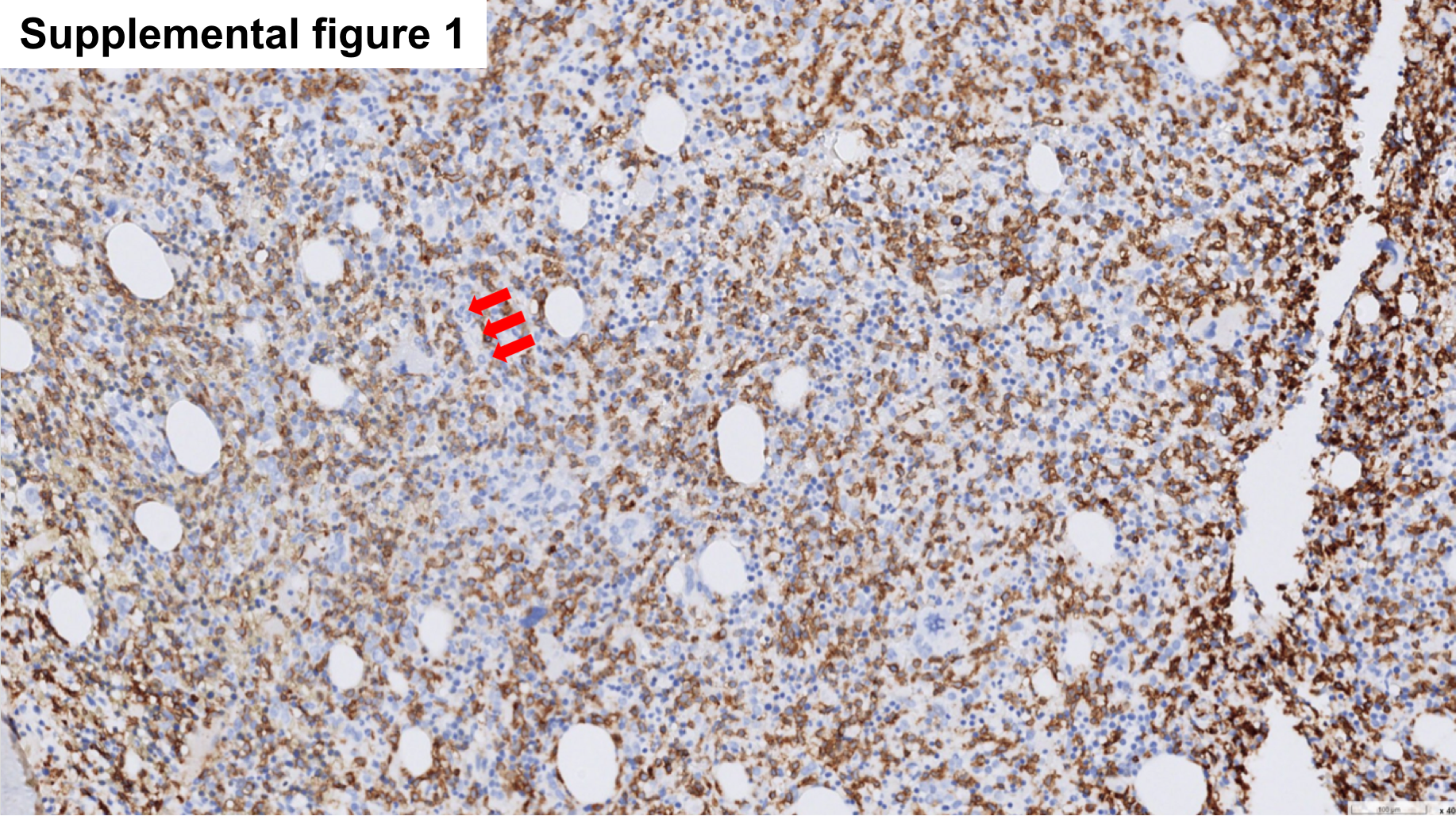

Supplement: Supplementary file 1 — Supporting Information [file JHA2-4-505-s001.png]
